# Direct Extraction of Lipids, β-Carotene, and Polyphenolic Compounds from Wet Microalga *Dunaliella salina* by Liquefied Dimethyl Ether

**DOI:** 10.3390/md22100438

**Published:** 2024-09-26

**Authors:** Hideki Kanda, Kaito Kusumi, Li Zhu, Tao Wang

**Affiliations:** 1 Department of Chemical Systems Engineering, Nagoya University, Chikusa, Nagoya 464-8603, Japan; 2 Department of Materials Process Engineering, Nagoya University, Chikusa, Nagoya 464-8603, Japan

**Keywords:** antioxidant, carotenoid, green solvent, microalgae, subcritical fluid

## Abstract

Extraction of lipids and high-value products from highly wet microalgae requires significant energy for the drying pretreatment. In this study, we examined the direct extraction of lipids, β-carotene, and polyphenolic compounds from wet *Dunaliella salina* using liquefied dimethyl ether (DME), which is effective in lipid extraction for biofuel production. The amount of DME-extracted β-carotene was 7.0 mg/g, which was higher than that obtained from the chloroform–methanol extraction. Moreover, the total phenolic content extracted with DME and its antioxidant capacity were slightly higher than those extracted with chloroform–methanol. DME removed almost all the water and extracted 29.2 wt% of total lipids and 9.7 wt% of fatty acids. More lipids were extracted from wet samples by liquefied DME than by chloroform–methanol extraction. The C/N ratio of lipids extracted with DME was 112.0, higher than that of chloroform–methanol. The high C/N ratio suggests that nitrogen-containing phosphatidylcholines may be less easily extracted by liquefied DME and may be highly selective. However, the ratio of saturated fatty acids was 34.8%, lower than that of chloroform–methanol. Na^+^ and Mg^2+^ in the culture medium were not extracted using DME. Thus, using the extract with DME has both advantages and disadvantages compared to using the extract with chloroform–methanol; however, it has satisfactory extraction properties. DME is expected to be an environment-friendly alternative solvent because it does not require drying, which is necessary for conventional extraction solvents.

## 1. Introduction

Carotenoids are natural hydrophobic pigments with bright yellow-to-red colors produced by plant synthesis. β-Carotene, a type of carotenoid, is abundant in microalgae [1,2] and is used as an antioxidant to remove active oxygen and as a natural coloring agent [3]. For extraction of these compounds, chloroform–methanol mixtures are frequently used. These mixtures of solvents are also used to extract lipids with a wide range of polarities and diversity of structures [4,5,6,7,8]. However, the use of toxic solvents during food processing remains challenging. A shift from organic solvents to green solvents has occurred owing to environmental and health concerns. For example, after extracting β-carotene using supercritical CO_2_ as a green solvent [8], the extracted β-carotene can be encapsulated in liposomes [9] using supercritical CO_2_ as a medium or β-carotene suspension [10]. 

Microalgae can be produced in land that has been abandoned for cultivation and they are attracting attention because of their high capacity to synthesize lipids and value-added compounds. Microalgae are suitable organisms for capturing atmospheric CO_2_ and implementing bio-cycle approaches [2]. *Dunaliella salina* is a microalga with a high β-carotene content. Several studies have used various solvents to extract β-carotene from *D. salina*. For example, acetone [11], a chloroform–methanol mixture [12], supercritical CO_2_ [13,14], and ethanol cosolvents [15,16] have been studied to extract β-carotene from *D. salina*. Microwave and ultrasonic pretreatment are also considered effective in the extraction of β-carotene from *D. salina* [17]. Microwaves not only break the cell wall [17], but also *Z*-isomerize the carotenoid [18], making it less crystalline [19] and thus easier to extract [20]. Ultrasonic treatment has also been confirmed to be effective in extracting β-carotene from *Spirulina platensis* [21]. However, these pre-treatments consume electrical energy, which increases the cost of the extraction. In the case of subcritical water extraction, the main objective is to hydrolyze *D. salina* into glucose and not to utilize it as lipid [22]. 

In the case of supercritical CO_2_ extraction, because β-carotene and supercritical CO_2_ are hydrophobic [2], the presence of coexisting water is a major problem when they are extracted from wet *D. salina*; thus, *D. salina* is dried as a pretreatment for extraction. For example, in the case of supercritical CO_2_ extraction, a sample with a moisture content of only 27% is considered sufficiently wet. This indicates that even the generally well-dried 27% moisture content is a harsh condition for supercritical CO_2_ extraction with high moisture content. When microalgae are recovered by centrifugation, the moisture content can be as high as 90%, which is a significantly different condition [23]. If the energy input for drying is derived from fossil fuels, CO_2_ emissions will increase, and the advantages of microalgae’s high β-carotene synthesis and CO_2_ absorption capacity will be lost. If energy is derived from green power, the high price and energy consumption of green power can be transferred to β-carotene production, resulting in inefficient production. Furthermore, hot air drying of microalgae degrades several microalgae components, not only in carotenes and phenolic compounds [24]. Therefore, although drying is necessary for β-carotene extraction, this step is a major problem from an environmental standpoint. Another problem is that the operating pressure required to process β-carotene using supercritical CO_2_ is very high, which compromises production costs and output.

To extract β-carotene from *D. salina* without drying, an amphiphilic solvent was used. However, when ethanol, a typical amphiphilic solvent, was used, the extract consisted of a mixture of water and lipids, including β-carotene. Because ethanol forms azeotropes with water, benzene is added to recover ethanol, which contaminates water and lipids. In addition, a large amount of distilled energy is consumed. In solving problems such as those encountered with ethanol, solvents with a much lower boiling point than water can be easily separated from water [8]. Furthermore, the solvent must be environmentally friendly, leave little residue in the water after evaporation, be nontoxic, and be safe for use in food processing. Because it fulfills these requirements, liquefied dimethyl ether (DME) has been proposed as a substance [8].

In recent years, DME has attracted attention as a novel green solvent. DME differs from common ethers (e.g., ethyl ether). Owing to its molecular structure, namely, because its normal boiling point is as low as −24.8 °C and it is a gas in its standard state, DME must be pressurized to 0.59 MPa before using it as a liquid solvent at 25 °C [25]. This low boiling point ensured that no residues remained in the extract [26]. Additionally, aqueous solutions of DME have been confirmed to be non-toxic to microorganisms in bioassays [26]. Furthermore, because DME is weak polar and forms weak electrostatic intermolecular interactions analogous to hydrogen bonds [27], liquefied DME is partially miscible with water [28,29,30]. Because of these physicochemical properties, liquefied DME can diffuse through the water surrounding the high-water-content material and contact the extraction target when used as the extraction solvent. One drawback of liquefied DME extraction is that the operating pressure is higher than atmospheric pressure, resulting in higher equipment costs compared to extraction using conventional organic solvents. 

**Figure 1:**
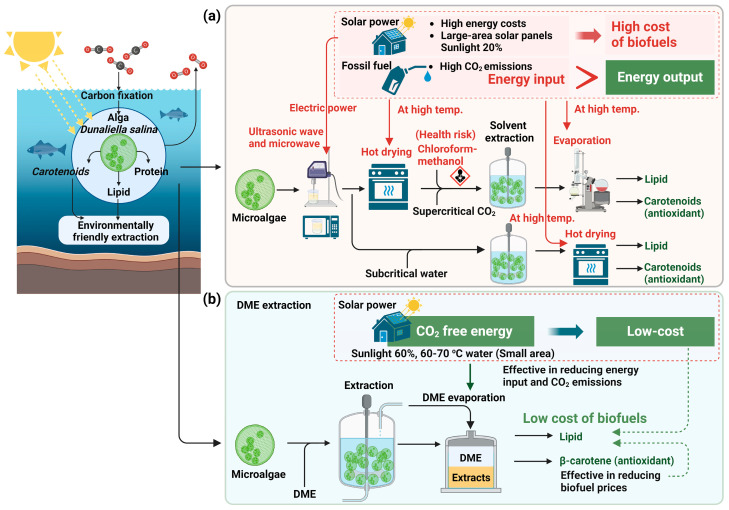
Procedure for extraction from microalgae: (**a**) previous studies, (**b**) this study.

When conventional organic solvents with high boiling points are used, expensive green electricity with low energy conversion efficiency from sunlight and fossil fuels with CO_2_ emissions are consumed for drying and boiling the organic solvents, as shown in Figure 1a. The energy conversion efficiency of photovoltaic power generation from sunlight to electricity is only about 20% or more, but in the case of solar hot water, it can reach 60%, as shown in Figure 1b. Moreover, solar hot water is inexpensive. In other words, in the case of liquefied DME, if solar hot water is used as a heat source for the evaporation of liquefied DME, the land required for its acquisition is smaller than that for photovoltaic power generation, and the energy input is less expensive [26]. However, the operating pressure for carotenoid extraction using supercritical CO_2_ is generally 20–30 MPa [13,14,15,16,20,31], and the equipment cost for DME extraction is lower than that for supercritical CO_2_. In other words, the adoption of liquefied DME as a new green solvent can fundamentally solve the equipment cost problem of supercritical CO_2_, which is a conventional green solvent, and expand the use of green solvents.

In addition, DME is environmentally friendly. It is a synthetic fuel with low greenhouse gas emissions and can be efficiently synthesized from biomass [32]. DME can also be catalytically and reductively produced from carbon dioxide by using green electricity [33]. The European Union has approved DME as an extraction medium [34], and the US Food and Drug Administration has classified DME as generally recognized as safe (GRAS) [35]. Additionally, unlike other alkyl ethers, the autoxidation of DME is comparable to that of liquefied petroleum gas (LPG) and DME can be safely handled using LPG handling techniques commonly used in the industry [36]. Therefore, liquefied DME, which avoids the air-drying of microalgae and use of traditional toxic solvents during extraction, offers an effective solution for carotenoid extraction. Studies have reported that carotenoids such as lutein [37] and fucoxanthin [26,38] can be extracted with liquefied DME without drying high-water-content algae. However, all carotenoids extracted in these previous studies contained hydroxyl groups. As mentioned earlier, DME forms weak electrostatic intermolecular interactions; therefore, it is easy to conclude that its interaction power with the substances extracted in previous studies is sufficient. However, β-carotene does not have hydroxyl groups, and there are no theoretical or experimental studies on its intermolecular interactions with DME. Therefore, it is unclear whether β-carotene can be extracted from liquefied DME. Thus, although β-carotene is a typical carotenoid and liquefied DME is effective for its extraction from microalgae with high water content, the extraction of β-carotene with liquefied DME has not been investigated. If lipids, β-carotene, and polyphenols could be extracted with liquefied DME, it would provide a means of processing *D. salina*, a type of microalga with excellent photosynthetic capabilities, using less energy and with higher efficiency than conventional solvents. This study investigated the possibility of extracting β-carotene and antioxidants from *D. salina* using liquefied DME, a suitable solvent for lipid extraction, for the effective utilization of microalgae. This study should provide a unified insight into the extractability of useful substances from microalgae in liquefied DME by elucidating whether β-carotene can be extracted by liquefied DME, in addition to the major carotenoids already identified, such as astaxanthin, fucoxanthin, and lutein. This should provide unified knowledge on the extractability of useful substances from microalgae in liquefied DME. This finding gives a means of processing microalgae under less CO_2_ emissions and energy consumption.

## 2. Results and Discussion

### 2.1. Basic Extraction Behavior

**Figure 2:**
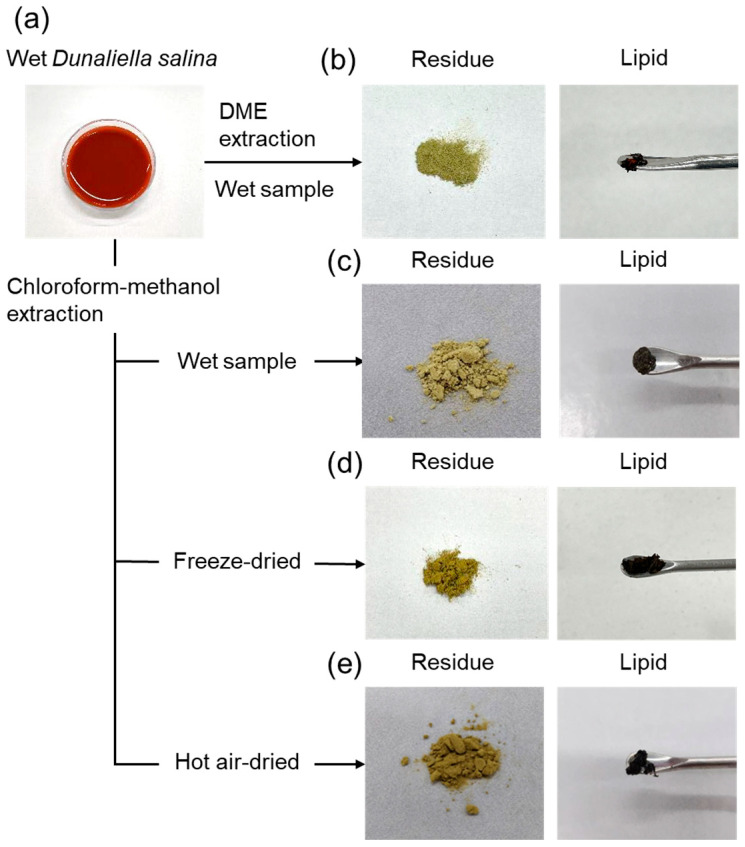
Images of (**a**) wet *D. salina*, (**b**) residue and lipid obtained from wet sample by DME extraction, (**c**) residue and lipid obtained from wet sample by chloroform–methanol extraction, (**d**) residue and lipid obtained from freeze-dried sample by chloroform–methanol extraction, and (**e**) residue and lipid obtained from hot-air-dried sample by chloroform–methanol extraction.

The extraction was terminated when the color of the extract leaving the extraction column changed from dark red to clear and colorless, the color of the original liquefied DME, that is, when the total extraction time was 84 min and the total volume of DME flow was 554 g (839 mL). Figure 2 depicts the wet *D. salina* sample, the lipids, and residues after extraction with liquefied DME and chloroform–methanol. As shown in Figure 2a, the wet *D. salina* was red because of its high β-carotene content. The residue obtained after liquefied DME extraction was a yellowish-green dried powder, as shown in Figure 2b, which was different in color from that of the original *D. salina*, suggesting that β-carotene was extracted. The liquefied DME extract was black, as shown in Figure 2b. In contrast, the color of the residue extracted from wet, freeze-dried, and hot-air-dried samples with chloroform–methanol was slightly darker than that extracted with liquefied DME, as shown in Figure 2c–e. Whether this phenomenon was due to insufficient extraction of β-carotene by chloroform–methanol or excessive extraction of other pigments by liquefied DME remains unclear. The color of the extract was black, as shown in Figure 2c–e, and did not differ in appearance from liquefied DME.

**Figure 3:**
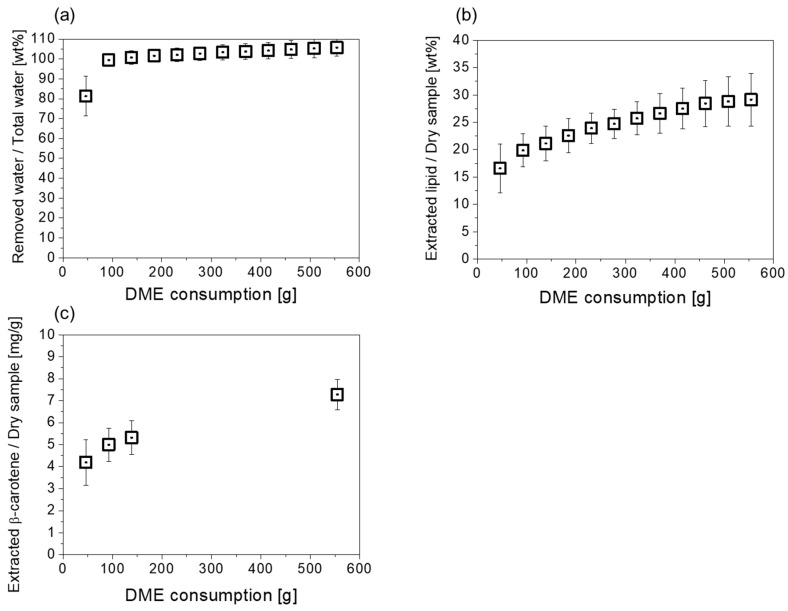
Changes in the amounts of materials extracted from wet *D. salina* by liquefied DME: (**a**) water; (**b**) lipid; (**c**) β-carotene.

Changes in water, lipids, and β-carotene extracted from wet *D. salina* using liquefied DME are shown in Figure 3a–c. Water extraction was almost complete initially. For example, 81.3 ± 10.0 wt% (=3.75 ± 0.46 g) of water was extracted when 46.2 g of liquefied DME flowed. This amount of water corresponds to 8.1 wt% of liquefied DME. As the saturation solubility of water in liquefied DME is 7.5 wt% [29], water was extracted with liquefied DME at a concentration almost equal to the saturation solubility, and some free water was pushed out of the column by the inflow pressure of the liquefied DME. This result is consistent with the free water unbound to the hydrophilic groups present in the cells, accounting for most of the water in *D. salina*. Thereafter, bound water, which was affected by hydrogen bonding with the hydrophilic groups of the cells, was extracted using liquefied DME. At 92.4 g of liquefied DME (which corresponds to DME/total water = 20.1), the extracted water was 99.4 ± 3.1%. For microalgae with a previous extracellular matrix structure, additional liquefied DME is required for water extraction. In other words, the curve showing the water extraction behavior of *D. salina* indicates that water can be extracted smoothly using less liquefied DME. For example, seven microalgal samples, including cyanobacteria and natural blue-green algae, showed approximately 50–70% of the total water extraction at liquefied DME/total water = 20 [8]. This difference may be due to *D. salina* not having a distinct extracellular matrix structure. If the extracellular matrix has pores and the pore walls are hydrophilic, interaction forces with water molecules occur. Theoretical predictions suggest that when a solute undergoes strong intermolecular interactions with a solid, the saturation solubility of the solute in the solvent is significantly reduced [26], and the presence or absence of this phenomenon may be related to differences in the water extraction behavior. When 554.4 g liquefied DME finally flowed, 105.7 ± 4.4% of the total water was extracted. This finding indicates that moisture that could not be removed by normal drying was also extracted using liquefied DME. In previous studies, water hydrogen bonded to the carboxyl group of sodium polyacrylate, the component of superabsorbent polymer, was extracted [39]. In other words, it is thought that the water that was not removed by normal drying was extracted by liquefied DME.

As shown in Figure 3b, 16.6 ± 4.5 wt% of the dry weight of *D. salina* was extracted as lipids when 46.2 g of liquefied DME was added. Thereafter, the amount of extracted lipids increased slowly until the end of the extraction when 554.4 g of liquefied DME flowed. Finally, the total amount of lipid extracted from *D. salina* with liquefied DME was 29.2 ± 4.8% of the dry weight of *D. salina*. In contrast, as shown in Table 1, the lipids extracted with chloroform–methanol were 20.1 ± 1.0% from the wet samples, 28.7 ± 1.6% from the freeze-dried samples, and 27.3 ± 1.8% from the hot-air-dried samples. This means that the performance is reduced when chloroform–methanol extraction is applied to wet samples. Figure 3c shows the changes in the amount of β-carotene extracted from *D. salina* using liquefied DME. As shown in Figure 3b, the small differential amount of extracted lipids after the fourth point of Figure 3b (after 184.8 g of liquefied DME) made quantitative analysis of β-carotene difficult; thus, all lipids after that point were collected together in one sample for analysis. The sum of the increments of β-carotene amounts in the region from the fourth point to the ninth point in Figure 3b was reflected in the last point of Figure 3c. At the end of the extraction, the β-carotene extracted with liquefied DME was 7.0 ± 0.7 mg/g of the dry *D. salina.* In contrast, as shown in Table 1, the β-carotenes extracted with chloroform–methanol were 1.8 ± 0.2 mg/g from the wet sample, 4.4 ± 0.8% from the freeze-dried sample, and 1.8 ± 0.3% from the hot-air-dried sample. This means that in the case of chloroform–methanol extraction, β-carotene yields are significantly decreased if samples are wet or hot-air-dried, and β-carotene yields are slightly decreased even when freeze-drying is applied to samples. In other words, liquefied DME is an extremely useful solvent that can be extracted from wet samples. Following these results, subsequent analyses of samples by chloroform–methanol extraction were performed on freeze-dried samples, which showed the best lipid and β-carotene extraction performance.

The slope of the first plot in Figure 3c shows the solubility of β-carotene in liquefied DME, 0.091 ± 0.022 mg/g of liquefied DME. This value is lower than the saturated solubility of 0.15–0.20 mg/mL (=0.23–0.30 mg/g) in liquefied DME reported previously [40]. Although *D. salina* has no distinct cell wall, the results still indicate that the cell membrane and coexisting water prevent the extraction of β-carotene, which does not have hydroxyl groups. Unlike easily extracted water, lipids and β-carotene are present in the cells of *D. salina* and are bound by intracellular intermolecular interactions. The solubility of solutes bound by intermolecular interactions with solids in the solvents decreased [26]. Additionally, the preferential extraction and loss of water between the cells of *D. salina* may have narrowed the distance between the cells of *D. salina*, creating resistance to mass transfer and requiring additional solvents to extract these substances [41].

**Figure 4:**
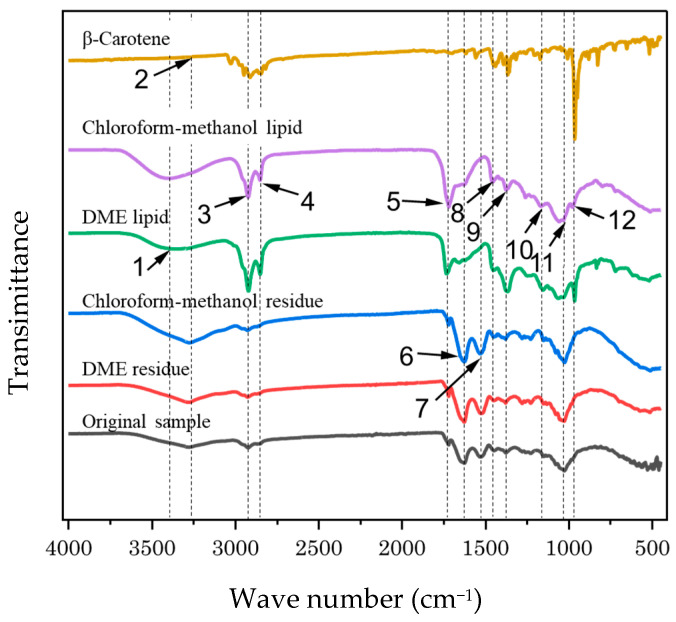
FT-IR spectra of the original sample (dry *D. salina*) and its residues and extracts after liquefied DME and chloroform–methanol extractions. The numbers correspond to the peak numbers listed in Table 2.

Figure 4 compares the Fourier transform infrared spectroscopy (FT-IR) spectra of lipid and residue obtained by liquefied DME from wet samples and by chloroform–methanol from freeze-dried samples. The detected peaks, functional groups suggested by the peaks, corresponding substances, and samples for which the peaks were detected are summarized in Table 2. Characteristic peaks 9 [42] and 12 [43], found only in β-carotene, were detected in both the liquefied DME and chloroform–methanol extracts. These two peaks were detected more strongly in the liquefied DME extract, indicating that more β-carotene was extracted from liquefied DME than from chloroform–methanol, as previously mentioned. β-Carotene peak 12 was buried by a large peak 11, representing polysaccharides [44], in the residues and the original sample, and the detection of peak 12 was unknown. β-Carotene peak 9 was detected as a significantly smaller peak in the residues and the original sample than in the extracts, which suggests that most of the β-carotene was extracted using both solvents.

Peaks 1 and 5 are characteristic of lipids [45,46], whereas peak 8 is derived from the functional groups present in β-carotene and lipids. These characteristics were detected with strong peak intensities in both extracts but with only slightly smaller peaks in both the residues and the original sample. This change suggests that both solvents extracted the lipids well. Peaks 6 and 7 are characteristic of proteins [45,46] and were detected in both the residues and the original sample but not in either extract. This suggests that the protein was not extracted using either solvent. This finding is consistent with that of the elemental ratio analysis using the CHNS-corder described in Table 3.

### 2.2. Polyphenolic Compound Characterizations

**Figure 5:**
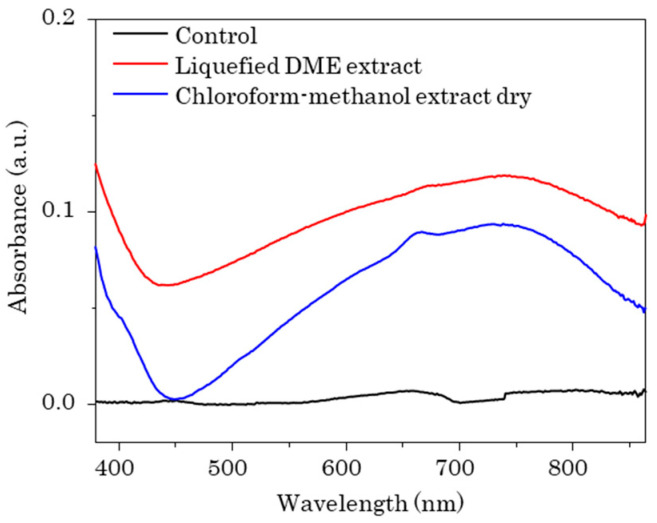
UV-Vis spectra in TPC content assay.

**Figure 6:**
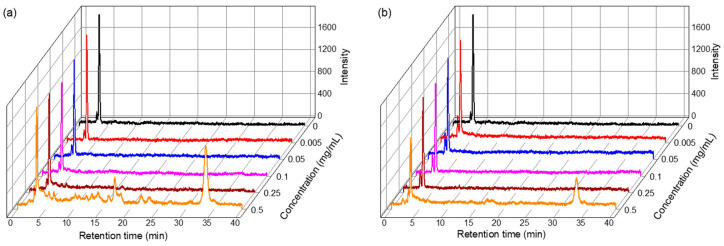
HPLC chromatograms in the DPPH radical scavenging activity assay: (**a**) DME extract, (**b**) chloroform–methanol extract.

As shown in Figure 5, the total polyphenolic content (TPC) was determined from the absorption intensity at 750 nm. The TPC with antioxidant activity was 6.11 ± 1.01 mg gallic acid equivalent (GAE)/dry-g of the algae sample for the liquefied DME extract and 5.59 ± 0.34 mg GAE/dry-g of the dry algae sample for the chloroform–methanol extract. As shown in Figure 6, the half-maximal (50%) inhibitory concentration (IC_50_) was measured from the absorption intensity at 517 nm. Peaks derived from di(phenyl)-(2,4,6-trinitrophenyl) iminoazanium (DPPH) were detected at 10 min in retention time and peaks derived from β-carotene at 34 min, confirming that other antioxidants and β-carotene were separated. As the amount of added lipids increased, DPPH was consumed by the antioxidants, decreasing the DPPH peak and increasing the amount of β-carotene detected. The IC_50_ was determined from the decrease in DPPH peaks at various lipid concentrations. In the DPPH radical scavenging activity assay, the IC_50_ was 0.66 ± 0.04 mg/mL-lipid for the DME extract and 0.73 ± 0.21 mg/mL-lipid for the chloroform–methanol extract. Comparing the TPC and IC_50_ of the lipids extracted with the two solvents, it is clear that the antioxidant capacity of the lipids extracted with liquefied DME was higher than that of the lipids extracted with chloroform–methanol. 

### 2.3. Lipid Characterizations

In addition to food oils and functional substances such as β-carotene, microalgae-derived lipids are used as biofuels. Thus, the elemental composition ratios were analyzed to explore the possibility of biofuel conversion. As shown in Table 3, the elemental ratios determined by the CHNS-corder confirmed that the lipids were enriched in C and H. The residues were enriched in N and O. The lipid content was higher than that of the residues. This result is consistent with that reported in the literature on water blooms [8]. Next, comparing the lipids extracted using the two solvents, the ratio of C to H in the lipids was significantly higher during liquefied DME extraction, as evidenced by the significantly small *p*-values shown in Table 4. Here, the C/N ratio is considered an important indicator in the choice of biomass conversion technology: thermochemical conversion processes are more suitable when the C/N ratio is above 30, and biochemical processes are more suitable when the C/N ratio is below 30 [47]. The C/N ratio of the original *D. salina* was 6.6, indicating suitability for biochemical processing. In contrast, the C/N ratio of the lipids obtained by liquefied DME extraction was 112.0, indicating the use of a thermochemical conversion process. The C/N ratio of the lipids obtained by chloroform–methanol extraction was 70.0, indicating that, although it was worse than that obtained by liquefied DME extraction, the thermochemical conversion process was applicable. The high C/N ratio suggests that nitrogen-containing phosphatidylcholines may be less easily extracted by liquefied DME and may be highly selective. The ratio of oxygen was increased in lipids extracted with chloroform–methanol compared to lipids extracted with liquefied DME, suggesting that some of the fatty acids in the chloroform–methanol extract may have been oxidized by oxygen in the air. Because such fatty acid oxidation degrades the quality of edible oils, extraction by liquefied DME, which does not require drying *D. salina* or heating for DME removal, is suitable from a food processing perspective.

**Figure 7:**
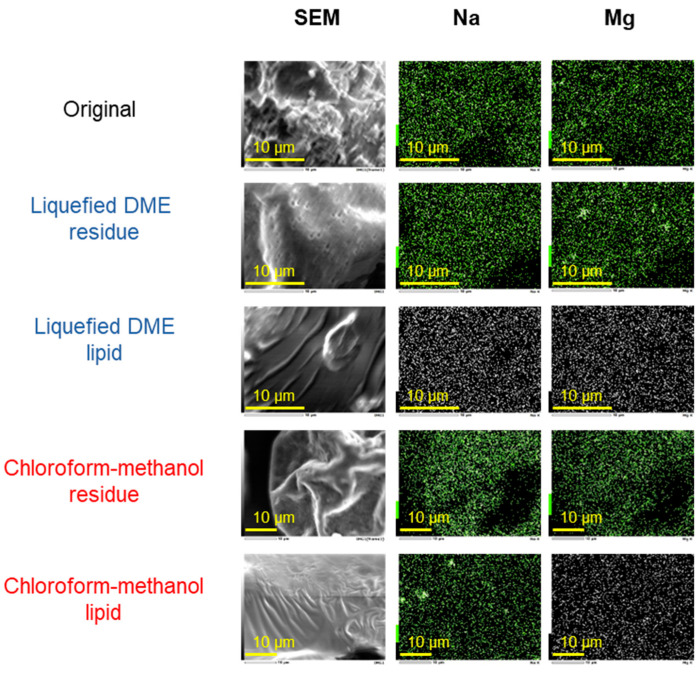
FE-SEM images of the original sample and its residues and lipids after liquefied DME and chloroform–methanol extractions.

Figure 7 shows field-emission scanning electron microscope (FE-SEM) images of the original *D. salina*, extracted lipids, and residues extracted using liquefied DME and chloroform–methanol. The constituent elemental ratios of the images are listed in Table 5. In the original *D. salina*, the Na and Mg contents were 0.43% and 0.23 %, respectively. Lipids extracted with liquefied DME contained 0.03 wt% Na and 0.02% Mg, whereas those extracted with chloroform–methanol contained 0.44 wt% Na and 0.05% Mg. Significant differences in Na and Mg content were observed between the two extraction methods for lipids, as indicated by the small *p*-values for lipids in Table 5. However, for Mg, the absolute amounts were low, near the detection limit; therefore, care should be taken regarding the interpretation of differences. Na^+^ was present in the chloroform–methanol-extracted lipids. In other words, liquefied DME does not dissolve Na^+^ or Mg^2+^ ions, whereas chloroform–methanol dissolves Na^+^ ions, and Na^+^ is transferred to the extracted lipids. Another study showed that glycine, a water-soluble amino acid, is insoluble in liquefied DME [26], and that NaNO_3_ and MgSO_4_ are probably similarly insoluble in liquefied DME. This suggests that low-molecular-weight molecules, which are highly polar and easily soluble in water, are difficult to dissolve in liquefied DME.

**Figure 8:**
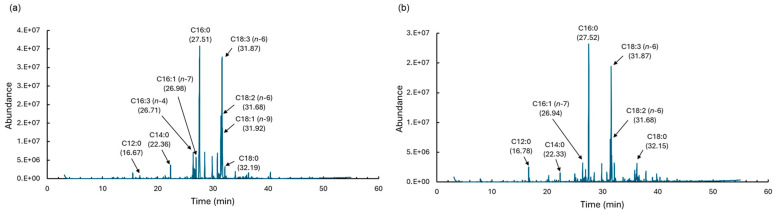
GC spectra of FAMEs: (**a**) DME extract, (**b**) chloroform–methanol extract.

Total fatty acid content was 9.7 ± 0.5 wt% of the dry weight of *D. salina* for the liquefied DME extract and 11.0 ± 0.3 wt% for the chloroform–methanol extract, indicating a slightly lower extraction rate of fatty acids with liquefied DME. Table 6 and Figure 8 shows the fatty acid composition of the lipids extracted from *D. salina* using these two solvents. Fatty acid methyl esters (FAMEs) comprising less than 1 wt.% of the total fatty acids in both solvents were excluded from the list. Fatty acids below 1 wt.% were also included in the sub-total saturated and unsaturated fatty acids. The fatty acid composition of the lipids extracted with liquefied DME was almost identical to that of the lipids extracted with chloroform–methanol, with C16 and C18 as the main components. Table 6 shows that the major fatty acid components of the extracted lipids were C18:3 (*n*-6), C16:0, and C18:2 (*n* − 6), with contents of 32.63–34.72%, 27.03–46.63%, and 12.98–14.31%. Of the three major fatty acids, C16:0 was significantly different by statistical analyses. The types of fatty acids present in high abundance were consistent with those reported for *D. salina* cultured under previously reported β-carotene-accumulating conditions [48]. As shown in Table 6, for the total amount of saturated fatty acids, the statistical analysis results indicated significant differences between the two extraction methods. The extracted saturated fatty acid contents were 34.76% for liquefied DME and 50.89% for chloroform–methanol, indicating that chloroform–methanol extraction is superior to liquefied DME extraction when the saturated fatty acid content suitable for biofuels is limited. Statistical analysis revealed significant differences in the total amounts of unsaturated fatty acids between the two extraction methods. Unsaturated fatty acids are desirable in food oils because of their low melting points and high antioxidant activities. However, the more double bonds in fatty acid, the more vulnerable it is to oxidation. In the case of chloroform–methanol extraction, which requires the drying of *D. salina* and evaporation of organic solvents at high temperatures, reactions with atmospheric oxygen are accelerated, resulting in acidified oil. The possibility of oxidation of unsaturated fatty acids during the extraction process is also strongly supported by the increased percentage of oxygen in the lipids extracted by chloroform–methanol, as shown in Table 3. The oxidation of unsaturated fatty acids is preferable to avoid the ingestion of volatile substances formed by acidification, as they may irritate the ocular mucosa or cause allergic reactions. It also impairs flavor by producing an offensive odor. Liquefied DME extraction avoids drying and evaporation of organic solvents at high temperatures, which is a possible reason for the increased percentage of unsaturated fatty acids detected. C18:3 (*n* − 6) detected in large amounts in the extracted lipids is γ-linolenic acid (ω-6 fatty acid), and C18:2 (*n* − 6) is linoleic acid (ω-6 fatty acid). ω-6 fatty acids are essential nutrients. Thus, lipids extracted using liquefied DME are suitable for the production of food oils. The lipid profile indicates value for food, or feed with a high relative content of ω-6 fatty acids. The fact that *D. salina* lipids contain high amounts of ω-6 fatty acids and are effective as food and feed was pointed out in a previous study [17], even with lipids extracted by conventional solvents. The results showed that modification of the lipid extraction method is also extremely effective in modifying the components contained in the lipids, especially the approach of using liquefied DME as an extraction solvent, which avoids drying and heating at high temperatures.

Chloroform–methanol, often used for batch extraction of lipids of various polarities, is suitable for extracting lipids, β-carotene, and polyphenolic compounds during microalgal food processing. However, the strong toxicity makes it unsuitable for food-processing purposes. Additionally, microalgae, which are attracting attention as environmentally friendly resources because of their high CO_2_ absorption capacity and water content, must be dried before use. However, drying is energy intensive; therefore, there is a dilemma between the significance of using microalgae and the need for drying. Another problem is that the functional ingredients are less stable during drying. Liquefied DME, a solvent that solves this problem, extracts β-carotene, polyphenolic compounds, and lipids in high yields from *D. salina* in its high-water content state. Thus, this solvent is promising for the effective utilization of microalgae without the need for energy-intensive drying pretreatments. Future research is needed to determine the effects of different extraction methods on the storage stability of extracts.

## 3. Materials and Methods

### 3.1. Materials

*D. salina* cultured in BG-11 culture medium (C3061, Sigma-Aldrich, St. Louis, MO, USA) was obtained from the Microalgae Corporation (Gifu, Japan). The water content was 90.0% after centrifugation, which was determined from the difference between the initial weight and the weight of the dried sample after heating to 107 °C to a constant weight. Liquefied DME (Air Can 420D; Tamiya, Inc., Shizuoka, Japan) was used for the extraction experiments without further purification. High-Performance Liquid Chromatography (HPLC)-grade chloroform and methanol as conventional solvents, β-carotene (90.0–102.0% [absorbance]), HPLC-grade acetonitrile and water, gallic acid (98.0–103.0%), sodium carbonate (99.8+%), and Folin–Ciocalteu reagents for the quantitative analysis of extracts were purchased from Fujifilm Wako Pure Chemical (Osaka, Japan). 

### 3.2. Liquefied DME extraction

**Figure 9:**
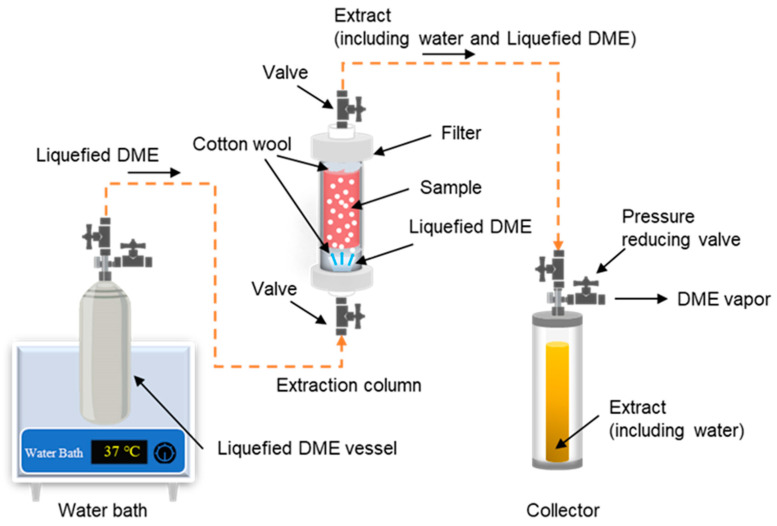
Schematic of liquefied DME extraction.

The DME extraction apparatus used in this study was similar to one reported in the literature [8]. The configuration of the apparatus is shown in Figure 9. First, a metal vessel (TVS-1; Taiatsu Techno, Saitama, Japan) containing liquefied DME was supplied, and an extraction column (HPG-10-5, 10 mL, 190 mm × 11.6 mm, Taiatsu Techno) and an extract collector (HPG-96-3, 96 mL, Taiatsu Techno) were connected by steel use stainless SUS 316 (ISO 4401-316-00-I) tubes with an inner diameter of 1/16 inch through which liquefied DME flowed. The extraction column and collector were composed of pressure-resistant glass covered with clear polycarbonate and printed on a volume scale. The lower half of the extraction column (5 mL volume) was packed with 5.12 ± 0.05 g of wet *D. salina* after centrifugation, and cotton and colorless glass beads were placed in the cavity of the extraction column to prevent sample migration. A cellulose filter with a pore size of 0.20 μm (C020A013A, Advantec Toyo, Tokyo, Japan) was placed at the outlet of the extraction column to prevent the sample from flowing out. By visually observing that β-carotene colored the liquefied DME, it was determined that β-carotene had been extracted, and that the extraction was terminated when the liquefied DME became colorless.

The metal vessel containing liquefied DME was heated in a water bath to 37 ± 1 °C to increase the saturated vapor pressure of the liquefied DME inside to 0.82 ± 0.02 MPa. Because the internal pressure of the extractant collector is relatively low, the liquefied DME flows under differential pressure. When the liquefied DME passed through the SUS316 connecting tube, the temperature decreased rapidly to 25 °C due to the large specific surface area of the connecting tube, which was confirmed by a non-contact infrared thermometer. The flow rate of the liquefied DME was adjusted to 10 ± 1 mL min^−1^ (=6.61 g min^−1^) [25] by manually adjusting the valve on the connecting tube based on the volume scale on the collector of the extracted solution. Each time the volume of extract in the collector was increased, the collector was immediately replaced with an empty collector. The end valve of the collector, removed from the apparatus, was opened, the pressure was reduced to atmospheric pressure, DME was evaporated, and the amount of DME was determined from the weight difference. The collector contained water and an organic extract containing β-carotene, and the weight of the extracted water was determined by the difference in weight after lyophilization. The organic extracts were collected in an 80:20 (*v*/*v*) acetonitrile/water mixture and used for subsequent HPLC analysis. To ensure reproducibility, the entire experiment, from extraction to analysis, was repeated thrice, and three deviations were recorded as errors.

### 3.3. Chloroform–Methanol Extraction

The basic solvent of the Folch method [5], a chloroform–methanol (2:1, *v*/*v*) mixture, was mixed with a wet sample in the same solvent ratio as for liquefied DME extraction. In other words, 6.00 g of wet *D. salina* (water content: 90 wt%) was mixed with 984 mL of chloroform–methanol solvent. This corresponds to the result of a completed extraction from a 5.12 g wet sample with 840 mL (=554.4 g) liquefied DME. Experiments were also conducted with two dried samples for reference. The two dried samples were freeze-dried and hot-air-dried at 120 °C for 1 hour. A total of 6.00 g of wet *D. salina* and 0.60 g of dry *D. salina* were disrupted in the chloroform–methanol solvent by a hand-held homogenizer equipped with a sawtooth generator probe (Dremel 300 Series, Robert Bosch Tool, Mount Prospect, IL, USA; 10mm for outside diameter). Stirring extraction was performed at room temperature for 2.5 h, and the residue was separated with a polytetrafluoroethylene filter with a pore size of 0.10 μm (H010A047A, Advantec Toyo). Subsequently, the chloroform–methanol solvent was evaporated using a rotary evaporator (SB-1200, Eyela Co., Ltd., Tokyo, Japan). Next, the influence of light on the decomposition of β-carotene was eliminated by covering the food mill and extractor with aluminum foil.

### 3.4. Analytical Methods

#### 3.4.1. β-Carotene Determination

The amount of β-carotene in the extract was measured using an HPLC system consisting of a deaerator (DG-980-50; Jusco, Tokyo, Japan), a pump (PU-980; Jusco), and a column heater (U-620; Sugai Chemie, Wakayama, Japan) and an ultraviolet–visible (UV-Vis) detector (UV-970, Jusco) controlled by Jusco Bowin (Ver 1.5) software via an LC-Net II/ADC controller (Jusco). β-Carotene was isolated at 40 °C by using an Inertsil ODS-3 column (250 mm × 4.6 mm × 5 μm, GL Science, Tokyo, Japan) with acetonitrile, with water (80/20 (*v*/*v*)) eluent flowing at a rate of 1.0 mL/min. The UV-Vis detection wavelength was set to 445 nm [26].

#### 3.4.2. FT-IR Characterization

To verify whether β-carotene was transferred from the original *D. salina* to the liquefied DME extract, we examined the functional groups of the extracted lipids and residues using FT-IR (Spectrum Two, PerkinElmer Japan, Yokohama, Japan) and compared the data with those of the original dried *D. salina*. Some of the samples to be measured in this study were solids, and the presence of solvents was problematic. Therefore, the instrument selected for this analysis without solvent was the attenuated total reflection FT-IR apparatus. A list of IR signals corresponding to the typical functional groups and possible compounds in *D. salina* was obtained from another study [44]. Additionally, IR data were compared with the IR signals of β-carotene [42,47,49].

#### 3.4.3. TPC Assay

The TPC of the extracts was measured using the Folin–Ciocalteu assay as previously described [50]. First, 1.0 mL of the extracted sample was mixed with 5.0 mL of deionized water and 6.0 mL of 7.5% (*w/w*) sodium carbonate. After 10 min, the Folin–Ciocalteu reagent (0.5 mL) was added, and the mixture was stirred for 5 min. After incubation for 2 h in the dark at room temperature, the absorbance at 750 nm was measured using a UV-Vis spectrophotometer (V-550, Jasco), representing GAE (range, 20–100 mg/L) [26].

#### 3.4.4. DPPH Radical Scavenging Activity Assay 

The antioxidant capacity of the extract was tested using the DPPH radical scavenging activity assay. DPPH was separated from β-carotene by HPLC [51] because the β-carotene in the extract overlaps with the 517 nm absorption wavelength range required for measuring DPPH [51]. The pre-treatment method was as follows. The extract was diluted with methanol to a concentration range of 0.005–0.5 mg/mL. Next, 2.7 mL of DPPH solution (6 × 10^−5^ M in methanol) was added, stirred, and allowed to stand at room temperature in the dark for 1 h. This solution was then injected into the HPLC system by using the same equipment, column, eluent, temperature, and injection volume as those used for measuring β-carotene, as mentioned in Section 3.4.1. The DPPH radical scavenging activity was determined using Equation (1):

(1) $$\%\;DPPH\;radical\;scavenging\;activity=\left(\frac{A_{517}\;of\;control-{\;A}_{517}\;of\;sample}{A_{517}\;of\;control}\right)\;\times \;100\%$$

where *A*_517_ of the control is the absorbance of the control and reagent at a wavelength of 517 nm, and *A*_517_ of the sample is the absorbance of the sample that reacted with the reagent at this wavelength. By plotting the ratio of DPPH radical scavenging activity against concentration, the IC_50_, which is the concentration of the sample required for 50% DPPH radical scavenging activity, was determined [52]. 

#### 3.4.5. Elemental Analysis

To investigate the separation of lipids and proteins in *D. salina*, an elemental analyzer (2400 Series II CHNS/O Elemental Analyzer, PerkinElmer, Japan) was used to analyze the proportions of C, H, N, O, and S and the constituent elements of *D. salina* [26]. 

#### 3.4.6. FE-SEM Characterization

To verify whether the salts from the culture medium were incorporated into the extract, the elemental compositions of the original sample, extract, and residue were determined by FE-SEM (JSM-7500F, JEOL, Tokyo, Japan). The elements measured were Na and Mg in NaNO_3_ and MgSO_4_, which were the main components of the culture medium [53]. The samples were coated with osmium to a thickness of 10 nm using sputtering equipment (osmium plasma coater OPC-60A, Filgen, Nagoya, Japan) with an acceleration voltage of 20.0 kV. Five measurements were obtained and the maximum and minimum values were rejected, leaving only three central measurements. 

#### 3.4.7. Fatty Acids Analysis

To determine the percentage of fatty acids in the extract, fatty acids were converted to FAME by acid-catalyzed esterification using a fatty acid methylation kit (06482-04; Nacalai Tesque, Kyoto, Japan), followed by a FAME purification kit (06483-94; Nacalai Tesque). After this pretreatment, analysis was performed using gas chromatography–mass spectrometry (GC–MS; 7890A GC system and 5975C inert XL MSD with a triple-axis detector; Agilent Technologies Japan, Hachioji, Japan) under the following conditions: The temperature of the phenyl-arylene capillary column (HP-5MS; 30 m × 0.25 mm i.d., Agilent Technologies Japan) was first held at 100 °C for 5 min and then was increased to 300 °C at 4 °C min^−1^. The injector and detector temperatures were set at 250 °C, and the split ratio was 1:1 (helium gas flow rate 24 mL min^−1^, injection volume 1.0 μL). The peak areas and retention times of the detected FAMEs were compared with those of the FAME standard (Supelco 37 Component FAME Mix; Sigma-Aldrich, St. Louis, MO, USA) and the mass spectrum in the NIST mass spectrum database [26].

#### 3.4.8. Statistical Analysis

Differences in TPC, IC_50_, elemental composition, and fatty acid composition between the two extraction methods were statistically analyzed. Statistical analysis was performed using GraphPad Prism 9 software at a 5% significance level (*p* < 0.05) to evaluate the differences between the two extraction samples. A *t*-test was used for this purpose. If the *p*-value was less than 0.05, the null hypothesis was rejected.

## 4. Conclusions

In this study, liquefied DME was successfully used to extract lipids, β-carotene, and polyphenolic compounds from the highly wet alga *D. salina*. The HPLC results showed that the β-carotene was extracted in higher yields than with the chloroform–methanol mixture. Moreover, the total phenolic content extracted with DME and its antioxidant capacity were slightly higher than those extracted with chloroform–methanol. DME removed almost all the water and extracted 29.2 wt% of total lipids and 9.7 wt% of fatty acids. Total lipids were more abundant than fatty acids in chloroform–methanol. The C/N ratio of lipids extracted with DME was 112.0, higher than that of chloroform–methanol; however, the ratio of saturated fatty acids was 34.76%, lower than that of chloroform–methanol. Na^+^ and Mg^2+^ in the culture medium were not extracted using DME. Thus, using the extract with DME has both advantages and disadvantages compared to using the extract with chloroform–methanol; however, in general, it has satisfactory extraction properties. DME is expected to be an environment-friendly alternative solvent because it does not require drying, which is necessary for conventional extraction solvents.

## Figures and Tables

**Table 1:** Lipid and β-carotene contents of lipids after liquefied DME and chloroform–methanol extractions.

Solvent	Sample	Lipid (wt%) *	β-Carotene (mg/g) *
Liquefied DME	Wet	29.2 ± 4.8	7.0 ± 0.7
Chloroform–methanol	Wet	20.1 ± 1.0	1.8 ± 0.2
	Freeze-dried	28.7 ± 1.6	4.4 ± 0.8
	Hot-air-dried	27.3 ± 1.8	1.8 ± 0.3

* The reported error is the standard deviation (SD).

**Table 2:** List of detected FT-IR peaks of the original sample and its residues and extracts after liquefied DME and chloroform–methanol extractions.

Peak	Wave Number (cm^−1^)	Functional Groups	Compounds	Samples with Peaks Detected
1	3392	CH=CH stretching	Lipid	Chloroform–methanol extract DME extract
2	3281	O-H, N-H symmetric stretching	Protein Water	Chloroform–methanol residue DME residue Original sample
3	2923	C–H stretching	Lipid β-Carotene	All samples
4	2851	CH_3_, CH_2_ symmetry stretching	Lipid β-Carotene	Chloroform–methanol residue DME residue Original sample
5	1722	C=O stretching	Lipid	Chloroform–methanol extract DME extract
6	1633	C=O stretching	Protein	Chloroform–methanol residue DME residue Original sample
7	1530	Amide II C-N stretching N-H angular vibration	Protein	Chloroform–methanol residue DME residue Original sample
8	1458	CH_2_ symmetry in-plane variation, CH_3_ inverse symmetry variable angle	Lipid β-Carotene	All samples
9	1377	C–H stretching	β-Carotene	All samples
10	1164	C-O stretching	Lipid	Chloroform–methanol extract DME extract
11	1046	C-O symmetric stretching	Polysaccharides	All samples
12	965	C=C out of plane bending vibration	β-Carotene	Chloroform–methanol extract DME extract

**Table 3:** CHNS-corder elemental ratios of the original sample and its residues and lipids after liquefied DME and chloroform–methanol extractions.

Solvent	Sample	C (wt%) *	H (wt%) *	N (wt%) *	O (wt%) *^,^**	S (wt%) *	C/N (−)
	Original	51.4 ± 0.2	6.1 ± 0.1	7.8 ± 0.0	34.0 ± 0.0	0.8 ± 0.1	6.6
Liquefied DME	Residue	46.5 ± 0.1	6.1 ± 0.1	10.7 ± 0.1	35.9 ± 0.2	0.9 ± 0.0	4.5
	Lipid	72.8 ± 0.1	10.6 ± 0.2	0.7 ± 0.0	16.0 ± 0.1	0.5 ± 0.1	112.0
Chloroform–methanol	Residue	42.4 ± 0.1	6.8 ± 0.0	9.3 ± 0.1	40.9 ± 0.1	0.6 ± 0.0	4.5
	Lipid	59.7 ± 0.1	8.4 ± 0.2	0.9 ± 0.0	30.7 ± 0.2	0.3 ± 0.1	70.0
*p*-Value	Residue	0.0000	0.0033	0.0001	0.0000	0.0001	0.0043
	Lipid	0.0000	0.0002	0.0092	0.0000	0.2375	0.0001

* Dry ash-free (daf) basis. The error was reported as the standard deviation (SD); ** determined by difference.

**Table 4:** TPC contents and IC_50_ of lipids after liquefied DME and chloroform–methanol extractions.

Solvent	Sample	TPC (mg GAE/Dry-g) *	IC_50_ (mg/mL-Lipid) *
Liquefied DME	Wet	6.11 ± 1.01	0.66 ± 0.04
Chloroform–methanol	Freeze-dried	5.59 ± 0.34	0.73 ± 0.21

* The reported error is the standard deviation (SD).

**Table 5:** FE-SEM elemental compositions of the original sample and its residues and lipids after liquefied DME and chloroform–methanol extractions.

Solvent	Sample	Na (wt%) *	Mg (wt%) *
	Original	0.43 ± 0.04	0.23 ± 0.05
Liquefied DME	Residue	0.40 ± 0.14	0.34 ± 0.14
	Lipid	0.03 ± 0.01	0.02 ± 0.01
Chloroform–methanol	Residue	0.23 ± 0.02	0.35 ± 0.00
	Lipid	0.44 ± 0.01	0.05 ± 0.01
*p*-Value	Residue	0.810	0.484
	Lipid	0.004	0.051

* The reported error is the standard deviation (SD).

**Table 6:** Fatty acid compositions of the lipids after liquefied DME and chloroform–methanol extractions.

Fatty Acid	Liquefied DME (wt%) *	Chloroform–Methanol (wt%) *	*p*-Value
C12:0	1.56 ± 0.97	0.60 ± 0.40	0.3339
C14:0	1.73 ± 0.31	1.47 ± 0.31	0.3472
C16:0	27.03 ± 6.15	46.63 ± 5.00	0.0289
C16:1 (*n* − 7)	4.50 ± 0.34	2.36 ± 0.58	0.0169
C16:3 (*n* − 4)	5.80 ± 2.54	0.00 ± 0.00	0.0854
C18:0	2.38 ± 0.23	2.19 ± 0.53	0.4589
C18:1 (*n* − 9)	4.42 ± 0.41	0.00 ± 0.00	0.0157
C18:2 (*n* − 6)	14.31 ± 3.96	12.98 ± 1.79	0.6838
C18:3 (*n* − 6)	34.72 ± 3.57	32.63 ± 2.10	0.4836
Saturated fatty acid	34.76 ± 7.97	50.89 ± 4.70	0.0387
Unsaturated fatty acid	65.25 ± 11.44	49.11 ± 4.70	0.0387

* The reported error is the standard deviation (SD).

## Data Availability

The data presented in this study are available on request from the corresponding author.
